# Three-year survival and recurrence after first-ever stroke: the Joinville stroke registry

**DOI:** 10.1186/s12883-015-0317-1

**Published:** 2015-05-01

**Authors:** Norberto Luiz Cabral, Milena Muller, Selma Cristina Franco, Alexandre Longo, Carla Moro, Vivian Nagel, Rafaela B Liberato, Adriana C Garcia, Vanessa G Venancio, Anderson RR Gonçalves

**Affiliations:** Brazil. Clinica Neurologica de Joinville. Joinville Stroke Registry, University of Joinville, Rua Otto Boehn, 571/202, 89201-700 Joinville, SC Brazil

**Keywords:** Epidemiology, Incidence, Recurrence, Stroke classification, Survival

## Abstract

**Background:**

Data estimating the recurrence and risk of death are lacking in low and middle income countries, where two thirds of the stroke burden occurs. Previously we had shown that the incidence and mortality have been decreasing over the last 18 years in Joinville, Southern Brazil. In this study, we aim to determine the recurrence rates, survival rates and the cause of death in 3 years after their first-ever incident in a urban population-based setting.

**Methods:**

From the Joinville Stroke Registry, we identified all the cases of first-ever stroke that occurred from October 2009 to September 2010. Multiple overlapping sources of information were used to ensure the completeness of case identification. Patients were followed up prospectively at regular intervals from 30-days to 3 years after the index event. Kaplan-Meir and Cox proportional hazards were used to assess the cumulative risk of death and recurrence.

**Results:**

We registered 407 first-ever stroke patients. After 3 years, 136 (33%) had died. In the first year of stroke the risk of death was 28% (95% CI, 25 to 32). Beyond the first year, approximately 3 to 5% of survivors died each year. The cumulative risk of death in ischemic stroke (IS) subtypes was 3.6 higher for cardioembolic (CE) IS (hazard ratio 3.6, 95% CI, 2.1 to 6.4; p = 0.001) and 3.3 times higher for undetermined IS (HR 3.3, 95% CI 1.9 to 5.8; p = 0.001) compared to small artery occlusion IS. Over 3 years, the overall stroke recurrence risk was 9% (35/407). We found no difference in stroke recurrence risk between IS subtypes. Cardiovascular disease was the main cause of death all follow up time.

**Conclusions:**

Compared to other cohort studies conducted between 10 and 20 years ago in high-income countries, our recurrence rates and 3-year risk of death were similar. Among IS subtypes, we confirmed that CE has highest risk of death. The most common cause of death after a first-ever stroke is cardiovascular disease. This has implications for the uptake of current secondary preventive strategies and the development of new strategies.

## Background

Stroke is the second most common cause of death and the third most common cause of disability-adjusted life-years (DALYs) worldwide in 2010 [1,2]. Incidence rates, 30-day case-fatality data, stroke survival rates and recurrence risks are useful epidemiological data which help to understand how well our primary prevention, quality of hospital care and secondary prevention have been conducted [3]. The overall burden of disease data from the World Health Organization shows stroke is still in the top 6 based on disability adjusted life-years [4]. Therefore, a comprehensive secondary prevention campaign should target the prevention of stroke recurrence, vascular dementia and death [5,6].

Recurrence and survival rates are useful data to compare the natural history of a disease against the effect of therapeutic interventions. In community-based studies which included all major stroke types together, the 3-year cumulative risk of recurrent stroke has been reported to be varied from 6 to 25% [7-10]. The reported cumulative risk of death in population-based studies which included all stroke types ranged across the last three decades in high and middle-income countries. In the 1980s in the UK, the 1-year risk was 31% and the 5-year risk was 50% [7]. In the 1990s, in Australia, the 1-year risk was 38% and the 5-year risk was 60%; [8]. In the 2000s, in Belarus, the 1-year risk was 38% and the 5-year risk was 59% [11]. As far as we know, no prospective population-based study has been conducted in a South American country, where the stroke burden is huge.

As we had shown that the incidence of stroke, 30-day case-fatality and mortality had fallen by a third in Joinville, Southern Brazil in the last decade [12] (1995 to 2005), we now aimed to know the stroke recurrence behavior in our population. Therefore, we conducted a prospective community-based study of a cohort of patients after their first-ever stroke to determine the 3-year survival rates, levels of recurrence and the major causes of death in Joinville, Brazil.

## Methods

### Study design and population

This is a population-based prospective cohort study. Using the Joinville Stroke Registry, an ongoing population-based registry was established in 2005. We ascertained all the cases of first-ever stroke occurring among patients living in Joinville city, Southern Brazil, between October 1, 2009, and September 30, 2010. This cohort was then followed up until September 30, 2013. On the basis of intercensus data, the estimated population of the study area was 509,743 inhabitants who lived in area of 1130 km [2,13]. Joinville city has four hospitals, three intermediate care units and one public institutional care facility. All the hospitals have computed tomography (CT) services available on a 24-hour basis. Two thirds of the city population uses only the public health system, which consists of nine state-run health districts with 56 primary care facilities.

### Baseline assessment

The cohort methods have been described elsewhere [12]. In brief, we used multiple overlapping data sets including medical records from all city hospitals, death certificates, and outpatient monitoring in the state-run and private units (WHO STEPS) [14,15]. Three research nurses discussed all the stroke cases occurring in all the hospitals with a neurologist on a daily basis. On a weekly basis, a neuroradiologist unaware of patients’ symptoms analyzed all brain CT scans, magnetic resonance images and digital angiographies. Every month we analyzed all death certificates (DC) issued by the Municipal Department of Health. We initially selected all death certificates containing any references to the tenth revision of the International Classification of Diseases (ICD-10) codes related to stroke (I61 through I69) or any descriptions of cerebrovascular diseases, as well as those listing the death as being from an unknown cause (R99). The deaths of patients not identified on hospitals records were investigated through the evaluation of hospital medical charts and, when available, imaging examinations. Every week the state-run health units searched their electronic records for any stroke-related diagnoses listed in the ICD-10. This happened monthly at the institutional care facility. However, not all patients identified agreed to be admitted. Such patients were offered an appointment as outpatient (1 or 2 per month, usually minor strokes or a stroke mimics). These records were reviewed and when necessary each medical chart was late discussed with a neurologist (NLC). Private city physicians received a reminder sticker with each study contact.

### Stroke definition and cohort criteria

Stroke was defined according to World Health Organization (WHO) definitions [16]. Ischemic stroke subtypes were classified according TOAST classification [17]. The primary outcomes were death or stroke recurrence. We defined recurrent stroke as new stroke event, additionally requiring a period of neurological stability of ≥24 hours between index and recurrent stroke, and the exclusion of other potential causes of neurological deterioration [18]. Individuals who had suffered the second stroke event were again interviewed in order to identify the subtype of the stroke recurrence. All surviving patients were followed-up through telephone calls by a research nurse of the Joinville Stroke Registry at 1, 6, 12, 24 and 36 months after the event. They also had face-to-face meetings with their neurologist or general physician. We classified the causes of death using standardized diagnostic criteria for stroke [16], recurrent stroke [18], myocardial infarction and other vascular death, and nonvascular death (see definitions, below) [19].

### Cohort inclusion and exclusion criteria

We included all the cases of residents of Joinville, regardless of age, diagnosed with any type of first-ever ischemic stroke (IS), primary intracerebral hemorrhage (PIH) or subarachnoid hemorrhage (SAH). We also included residents in the city with confirmed strokes that occurred outside the city limits. Patients experiencing a stroke for the first time who had experienced a previous transient ischemic attack (TIA), were also classified as a first-ever stroke case. We excluded TIA diagnoses, all recurrent events, and stroke patients who died in the first 24 hours of onset, as well as patients residing outside the Joinville city limits, or patients with subdural, epidural or intracerebral hemorrhage secondary to arteriovenous malformation or tumors.

### Stratification types of deaths

We classified the causes of death into the 5 groups that were used in the Oxfordshire Community Stroke Project: [7] (1) deaths resulting from a first-ever stroke due to the direct effects of a brain lesion or complications of resulting immobility, including deaths from bronchopneumonia even if they occurred several years after the stroke if the stroke-related disability was thought to be contributory; (2) deaths resulting from recurrent stroke that were directly due to a brain lesion or complications of immobility; (3) deaths caused by cardiovascular events that were definitely or probably from myocardial infarction, ruptured aortic aneurysms, peripheral arterial disease, or sudden death when there was no alternative explanation; (4) deaths from nonvascular events that were not due to any stroke-related disability and included such illnesses as cancer, injuries, or suicide; and (5) deaths of undetermined cause where there was insufficient information to establish a cause.

### Statistical analysis

We performed descriptive statistical analysis to compare baseline characteristics using the qui-square test, the Student *t* test, or the Mann–Whitney test according to distribution. The Kaplan-Meier product-limit technique was used to generate survival probabilities and survival curves. The risk ratio was estimated by using the Cox proportional model and the Log-Rank test to compare survival curves among stroke types. We used the R Project for Statistical Computing program version 3.1.1. The study was approved by the ethics in research committees of the Hospital Municipal São José, the Hospital Regional, the Centro Hospitalar Unimed, the Hospital Dona Helena, and the University Hospital of Joinville Region-Univille, Brazil.

## Results

### Study population

**Figure 1:**
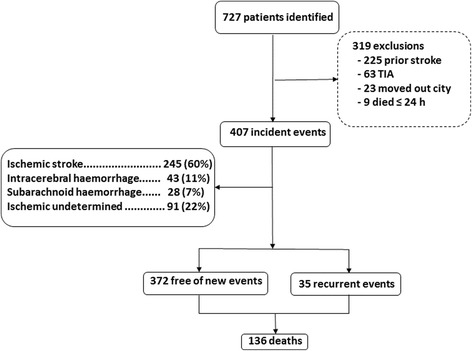
Flowchart of stroke recurrence or death over 3 years after first-ever stroke in Joinville, Brazil.

We identified 727 patients with stroke from October 1, 2009 to September 30, 2010. Of those, 225 had prior strokes, 63 were TIA events, nine patients died in the first 24 hours and 23 patients moved to other cities. These 320 patients were excluded. Therefore, the final cohort sample was 407 patients, among whom 83% (336/407) suffered ischemic strokes, 11% (43/407) had primary intracerebral hemorrhages and 7% (28/407) experienced subarachnoid hemorrhages (Figure 1).

The mean age of these patients at baseline was 63 ± 16 years, with a median age of 64 years. The ischemic stroke subtypes distribution by TOAST classification were: 27% (92/336) small artery occlusion (SAO); 27% (91/336) undetermined (UND); 25% (84/336) cardioembolic (CE); 17% (57/336) large artery atherothrombotic (LAA) and 4% (12/336) other determined strokes (OD). Table 1 shows the baseline characteristics, premorbid medications and premorbid risk-factor prevalence among IS, PIH and SAH patients.

**Table 1:** **Premorbid cardiovascular risk factors and medications in patients with first ever ischemic stroke, primary intracerebral hemorrhage and subarachnoid hemorrhage, Joinville 2009-2010**

	**IS (n = 336)**	**PIH (n = 43)**	**SAH (n = 28)**
**Baseline characteristics**			
Men	189 (56%)	19 (44%)	14 (50%)
Age, in years, mean (SD)	63 (16)	62 (16)	60 (13)
**Premorbid medication**			
Antiplatelet agent	102 (30%)	5 (12%)	3 (11%)
Anticoagulant	12 (4%)	2 (5%)	0 (0%)
Lipid lowering agent	59 (18%)	5 (12%)	6 (21%)
**Premorbid risk factor**			
Total cholesterol			
Mean baseline (mg/dl)	191 (46)	184 (30)	196 (57)
Proportion ≥ 6 mmol/L	45 (16%) [1]	2 (8%) [2]	2 (33%) [3]
Systolic blood pressure			
Mean (SD)	144 (27)	166 (36)	142 (25)
Proportion ≥ 150 mmHg	106 (39%) [4]	14 (56%) [5]	7 (32%) [6]
Proportion ≥ 160 mmHg	80 (29%)	9 (36%)	3 (14%)
Diastolic blood pressure			
Mean (SD)	87 (16)	95 (21)	88 (14)
Proportion ≥ 90 mmHg	69 (26%) [7]	10 (44%) [8]	6 (29%) [9]
Proportion ≥ 100 mmHg	30 (11%)	7 (30%)	1 (5%)
Smoking			
Current	67 (20%)	9 (21%)	12 (43%)
Ex	102 (30%)	14 (32%)	4 (14%)
Never	168 (50%)	20 (47%)	9 (32%)
Diabetes	78 (23%)	5 (12%)	2 (8%)
Atrial fibrillation	38 (11%)	5 (12%)	0 (0%)
Myocardial infarction	32 (9%)	2 (5%)	1 (4%)

IS: ischemic stroke; PIH: primary intracerebral hemorrhage; SAH: subarachnoid hemorrhage; Total cholesterol data were available in: [1] (286); [2] (26) [3] (6); Systolic blood pressure in: [4] (272) [5], (25) [6], (22); Diastolic blood pressure in: [7] (269) [8], (23) [9], (21).

### Outcome at 3 years

#### Absolute Risks for All Patients

**Figure 2:**
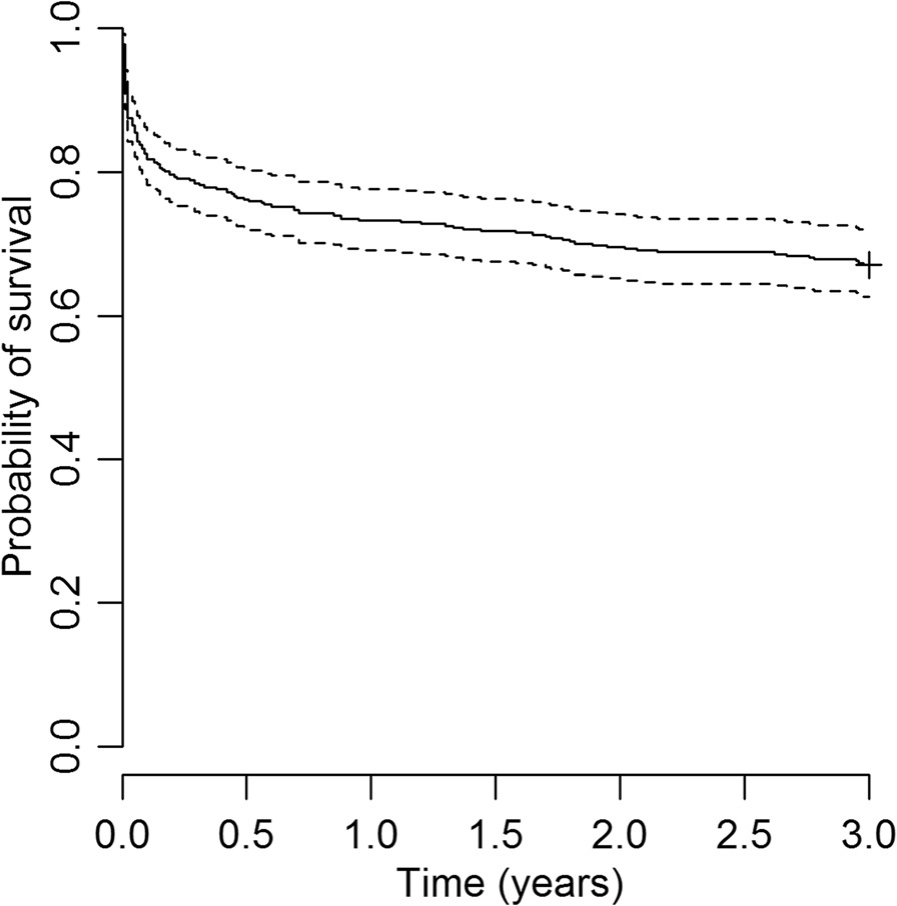
Kaplan-Meier curve showing the 3-year probability of survival after a first-ever stroke derived from Joinville Stroke Register, 2009–10. Dots on either side of the solid line indicate 95% CIs; n indicates number at risk at the beginning of each year.

Surviving patients were followed-up for a minimum of 3 years and up to 3 years and 11 months. Over 3 years of follow-up, 136 died and 35 had a recurrent stroke. Table 2 and Figure 2 shows that the 3-year cumulative risk of death was 33.4% (95% CI, 28.9 to 38.3). The risk of death was greatest in the first year after stroke (24.1%; 95% CI, 20.0 to 29.0) and particularly in the first 30 days after stroke (21.6%; 95% CI, 17.7 to 25.9). Beyond the first year, approximately 3 to 5% of survivors continued to die each year. No patient was lost to follow-up.

**Table 2:** **Kaplan-Meier estimates of probability of survival within defined time intervals after the index stroke**

	**1 day to 30 days**	**1 to 6 months**	**6 m to 1 year**	**1-2 years**	**2-3 years**
Risk of death %*	21.6	2.9	4.9	4.8	3.2
CI 95%	(17.7-25.9)	(1.4-5.4)	(2.7-7.9)	(2.7-7.9)	(1.5-6.0)
Cumulative risk, %	21.6	24.1	27.8	31.2	33.4
CI 95%	(17.7-25.9)	(20.0-29.0)	(24.5-32.3)	(26.7-36.0)	(28.9-38.3)
Number at risk	407	319	309	294	280
Number of deaths	88	10	15	14	9
Cumulative deaths	88	98	113	127	136

*Risk of death = (1-Kaplan-Meier estimate).

##### Absolute risks for subgroups

**Figure 3:**
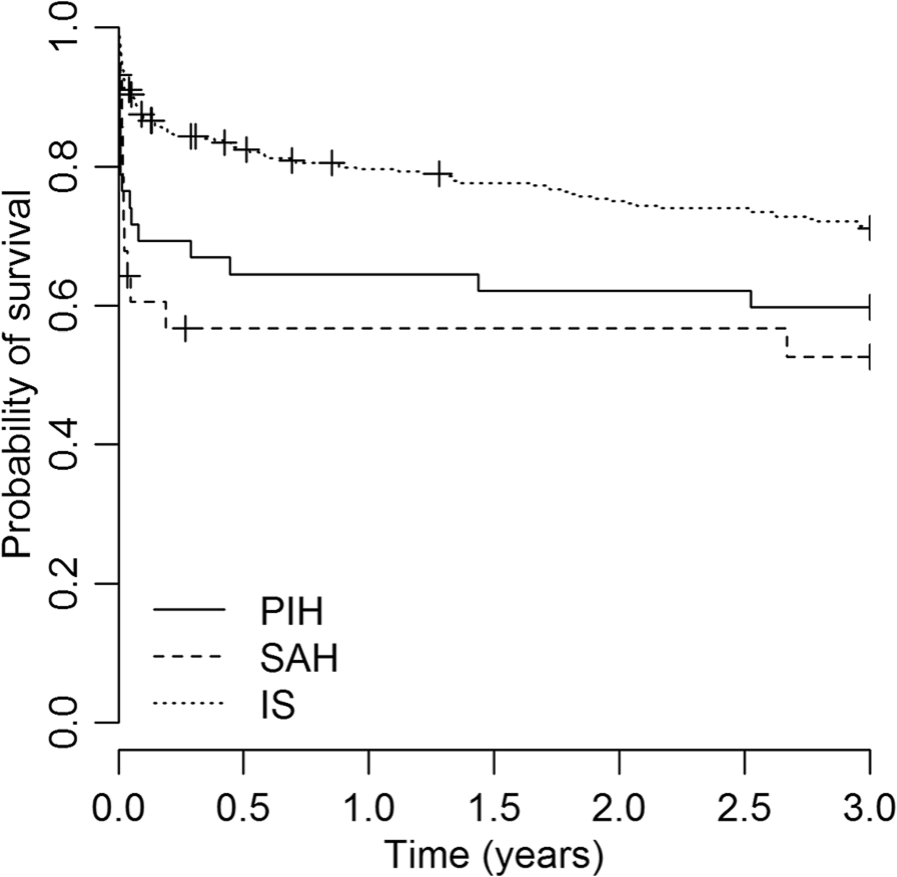
Kaplan-Meier curve showing the 3-year probability of survival after a first-ever stroke among primary intracerebral hemorrhage (PIH), sub-arachnoid hemorrhage (SAH) and ischemic stroke (IS).

As expected, stratification by the type of the first-ever stroke showed that subarachnoid hemorrhage was associated with a substantially greater number of case fatalities. The 30-day case-fatality rate was 54% (12/28) in SAH, 37% (16/43] in HS and 12% (40/336) in IS patients. After the first month the cumulative risk of death for all stroke types decreased. Up to 3 years the cumulative risk of death was 54% (15/28) in SAH, 53% (23/43) in PIH and 27% (92/336) in IS. Figure 3 compares the survival probabilities between IS, PIH and SAH.

**Figure 4:**
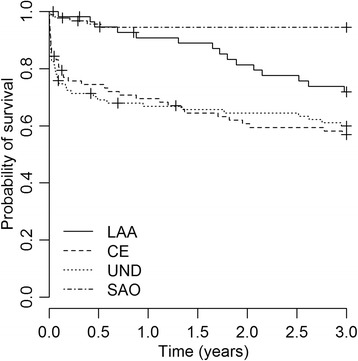
Kaplan-Meier curve showing the 3-year probability of survival after first-ever large artery atheroatherotsclerosis (LLA), cardioembolic (CE), undetermined (UND) and small artery occlusion (SAO) ischemic stroke.

Among IS sub-types, the 30-day case-fatality was 22% (20/91) in UND, 18% (15/85) in CE, 9% (1/11) in OD, 2% (2/92) in SAO and also 2% (1/57) in LAA. Figure 4 compares the survival curve among IS over 3 years (other determined data was not included). Compared to small artery occlusion, cardioembolic stroke carried a 3.6 times greater risk of death (HR 3.6, 95% CI, 2.1 to 6.4; p = 0.001) and undetermined stroke had a 3.3 times greater risk of death (HR 3.3, 95% CI 1.9 to 5.8; p = 0.001). Data are shown in supplementary Table 1.

**Figure 5:**
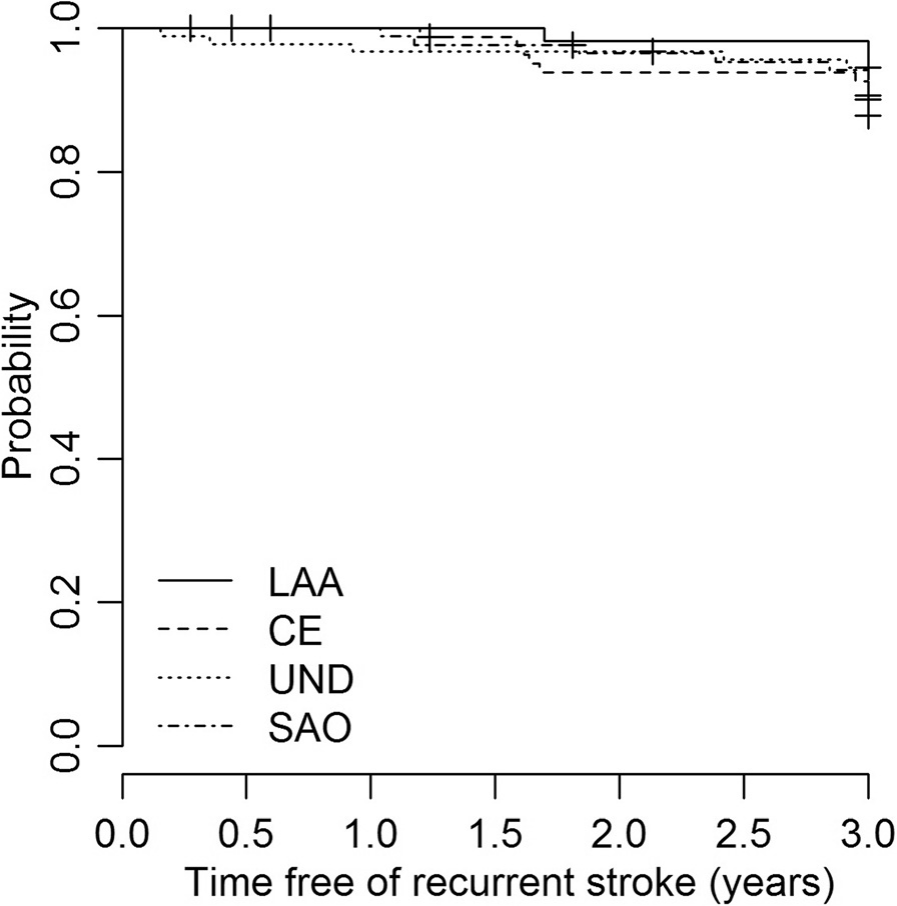
Kaplan-Meier survival curve showing the probability that, given survival, a patient with a stroke will remain free of a recurrent stroke, stratified by IS subtype (other determined IS not included).

Over three years we identified a cumulative risk of 9% (35/407) for stroke recurrence. Of those, 17% (6/35) died. We found no difference in stroke recurrence rates between IS subtypes (Figure 5).

**Figure 6:**
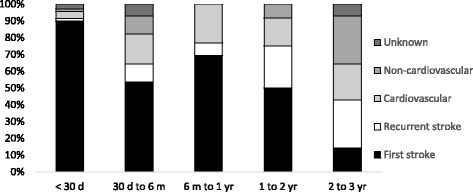
Histogram showing the proportion of patients dying from different causes during different time intervals from the onset of their first-ever stroke.

Figure 6 shows the causes of death at different time intervals after a first-ever stroke onset. During the first 30 days, 90% of deaths were due to the direct neurological effects of the index stroke. Among 30-day survivors up to 3 years, 71% of subsequent deaths were due to the first-ever (64%) or a recurrent (7%) stroke, and 23% were due to other cardiovascular causes. Among 3-year survivors, 43% of subsequent deaths were due to the first-ever (14%) or a recurrent (29%) stroke, and 7% were due to other cardiovascular causes.

## Discussion

We identified 407 first-ever stroke cases in 2009–10 in Joinville, Brazil. Over the next 3 years, the recurrence rate was 9%, with no difference in the recurrence risk among IS subtypes. As expected, the absolute risk of death was higher in the first 30 days (22%), decreasing to 3% to 5% in the following 3 years. Therefore, a sixth of patients died within 30 days and a third died within 3 years. The 30-day case-fatality rate consisted of more than a half of SAH patients, more than a third of PIH patients and 12% of IS patients. Despite the lower case-fatality rate among IS patients, the risk of death was very distinct among IS subtypes, where CE and undetermined IS had a worse prognosis than the other IS subtypes. Indeed, the 30-day case-fatality rate was 22% in undetermined IS and 18% in CE, whereas we found 9% in other determined IS cases and 2% in SAO and LAA strokes. As in other community-based studies [19-21] we also found CE and undetermined IS were the most lethal among IS subtypes. Three years after stroke onset, CE ischemic stroke patients had a 3.6 times higher risk of death, and undetermined IS patients had a 3.3 times higher risk of death than those with small artery occlusion (p < 0.001). The 136 deaths observed in the follow-up period included all causes of death. However, most of these were caused by cardiovascular complications in the brain or in the heart. Indeed, intracranial hypertension and immobility complications from the first-ever stroke, a recurrence, or an acute myocardial infarction were responsible for 90% of deaths within 30 days, 100% of those who died within a year and 50% of those who died within 3 years.

As far as we know, there are no other stroke survival or recurrence cohort studies in a community-based setting from low or middle-income countries. Our results were similar to other cohorts, which included all major stroke types conducted between the 1980s and the 2000s in high-income countries [8-11,22,23]. For example, in a meta-analysis with 9115 survivors from 13 studies, the pooled cumulative risk of stroke recurrence was 3.1% (95% CI, 1.7– 4.4) at 30 days, 11.1% (95% CI, 9.0 –13.3) at 1 year and 26.4% (95% CI, 20.1–32.8) at 5 years [24]. At 3 years, our recurrence rate was 8.6% (95% CI, 6.1-11.7). A cohort from Oxfordshire reported that the risk of early recurrent stroke was highest in patients with LAA, a finding that supports the need for urgent carotid imaging and prompt endarterectomy [24]. However, as in other studies we did not find a higher pattern of stroke recurrence among major stroke types or IS subtypes [9,11,19,20,23].

In Joinville, the one-year cumulative risk of death after a first-ever stroke was 28% (95% CI, 25% -32%) and 33% (95% CI, 29% -38%) after 3 years. Compared with other cohorts which also included SAH, PIH and IS in their samples, our rates are significantly lower than in Oxfordshire, UK (1-year: 44%, 95% CI 41-48%; 3-years: 50%, 95% CI, 46% -54%) [7] and in Perth, Australia (1-year: 43%, 95% CI, 38%-48%; 3-years:50%, 95% CI, 44% -54%) [9]. several authors have studied prognostic factors for long-time survival rates after stroke [10,25]. It is well stablished that old age and severe impairment after stroke predict poor outcomes [24,26]. We have not stratified our sample by stroke severity but one explanation for our lower risk of cumulative death might be related to lower patient age. The Joinville mean age was 63 (SD 16) years old, lower than 72 (SD not specified) years old in Oxfordshire and 73 (SD 13) years old and in Perth [7,8,12,22]. Therefore, the older age for first-ever onset in UK and Australia might contribute to a worse death rate in these populations, albeit the CI rates overlapped with our sample. Joinville has been using IS thrombolysis in most city hospitals since 2005 and in its stroke unit since 2000 [26,27]. Of course we cannot measure with accuracy how much these interventions, coupled with new drugs for secondary prevention which became available in the last 20 years, might be related to our better results.

There are limitations to our study. First, no patient agreed to an autopsy. Despite the fact that we double-checked each cause of death by phone and by death certificate analysis, we had no autopsy data. In contrast, the Hisayama cohort did an autopsy in 82% of their sample [28]. Although the proportion of cases was not specified, the Oxfordshire and Perth cohorts reported they also carried out autopsies [7,8]. Second, we believe that our data on the proportion of stroke recurrence might be underdetermined because it is possible that we missed some mild strokes that were not treated in hospitals. To avoid the capture of mild cases, we have been use the third step of WHO Stepwise surveillance criteria [14]. However, we believe that this strategy is inferior to the face-to-face follow-up meetings reported in Hisayama, South London, Oxfordshire, Perth, Erlangen and Melbourne studies [7,8,10,28,29]. Our strengths are the novelty of this data in low and middle-income countries. Our Registry is an ongoing state-run program so we have the ability to go back and measure the trends of recurrence and mortality risk in the near future in Joinville.

## Conclusion

Within a community-based study design, we have confirmed that our recurrence rate are similar to other population-based studies. Our cumulative risk of death is lower than other cohorts with the same inclusion criteria, which were however conducted between one and two decades ago. As with many other authors, we also found that CE and undetermined strokes have a worse prognosis than other IS subtypes. Further studies in populations with similar socioeconomic profiles need to be do done in order to compare our results and to help in planning health policies for secondary stroke prevention.

## Abbreviations

ICD-10: Tenth revision of the International Classification of Diseases
CT: Cranial tomography
DC: Death certificates
HR: Hazard ratio
IS: Ischemic stroke
PIH: Primary intracerebral hemorrhage
SAH: Subarachnoid hemorrhage
CE: Cardioembolic
SAO: Small artery occlusion
LAA: Large artery atherothrombotic
UND: Undetermined
OD: Other determined
TIA: Transient ischemic attack

## References

[1] Lozano R, Naghavi M, Foreman K, Lim S, Shibuya K, Aboyans V (2012). Global and regional mortality from 235 causes of death for 20 age groups in 1990 and 2010: a systematic analysis for the global burden of disease study 2010. Lancet.

[2] Murray CJ, Vos T, Lozano R, Naghavi M, Flaxman AD, Michaud C (2012). Disability-adjusted life years (DALYs) for 291 diseases and injuries in 21 regions, 1990–2010: a systematic analysis for the global burden of disease study 2010. Lancet.

[3] Feigin VL, Lawes CM, Bennett DA, Barker-Collo SL, Parag V (2009). Worldwide stroke incidence and early case fatality reported in 56 population-based studies: a systematic review. Lancet Neurol.

[4] Mathers C, Fat DM, Boerma JT (2008). The global burden of disease: 2004 update.

[5] Pendlebury ST, Rothwell PM (2009). Prevalence, incidence, and factors associated with pre-stroke and post-stroke dementia: a systematic review and meta-analysis. Lancet Neurol.

[6] Hankey GJ (2003). Long-term outcome after ischaemic stroke/transient ischaemic attack. Cerebrovasc Dis.

[7] Burn J, Dennis M, Bamford J, Sandercock P, Wade D, Warlow C (1994). Long-term risk of recurrent stroke after a first-ever stroke. The Oxfordshire Community Stroke Project. Stroke.

[8] Hankey GJ, Jamrozik K, Broadhurst RJ, Forbes S, Burvill PW, Anderson CS (1998). Long-term risk of first recurrent stroke in the Perth community stroke study. Stroke.

[9] Elneihoum AM, Göransson M, Falke P, Janzon L (1998). Three-year survival and recurrence after stroke in Malmö, Sweden: an analysis of stroke registry data. Stroke.

[10] Hillen T, Coshall C, Tilling K, Rudd AG, McGovern R, Wolfe CDA (2003). Cause of stroke recurrence is multifactorial: patterns, risk factors, and outcomes of stroke recurrence in the South London stroke register. Stroke.

[11] Kulesh SD, Kastsinevich TM, Kliatskova LA, Sauchanka ME, Filina NA, Shumskas MS (2011). Long-term outcome after stroke in Belarus: the Grodno stroke study. Stroke.

[12] Cabral NL, Gonçalves ARR, Longo AL, Moro CHC, Amaral CH (2009). Trends in stroke incidence, mortality and case fatality rates in Joinville, Brazil: 1995–2006. J Neurol Neurosurg Psychiatry.

[13] Instituto Brasileiro de Geografia e Estatística- IBGE. Brazil 2010 census. http://censo2010.ibge.gov.br/resultados. Accessed 02 April 2015

[14] Truelsen T, Heuschmann P, Bonita R, Arjundas G, Dalal P, Damasceno A (2007). Standard method for developing stroke registers in low-income and middle-income countries: experiences from a feasibility study of a stepwise approach to stroke surveillance (STEPS Stroke). Lancet Neurol.

[15] Sudlow CL, Warlow CP (1996). Comparing stroke incidence worldwide: what makes studies comparable?. Stroke.

[16] Aho K, Harmsen P, Hatano S, Marquardsen J, Smirnov VE, Strasser T (1980). Cerebrovascular disease in the community: results of a WHO collaborative study. Bull World Health Organ.

[17] Adams HP, Bendixen BH, Kappelle LJ, Biller J, Love BB, Gordon DL (1993). Classification of subtype of acute ischemic stroke. definitions for use in a multicenter clinical trial. TOAST. Trial of Org 10172 in acute stroke treatment. Stroke.

[18] Jackson C, Sudlow C (2005). Comparing risks of death and recurrent vascular events between lacunar and non-lacunar infarction. Brain.

[19] Leyden JM, Kleinig TJ, Newbury J, Newbury J, Castle S, Cranefield J (2013). Adelaide stroke incidence study: declining stroke rates but many preventable cardioembolic strokes. Stroke.

[20] Bejot Y, Caillier M, Ben Salem D, Couvreur G, Rouaud O, Osseby G (2008). Ischaemic stroke subtypes and associated risk factors: a French population based study. J Neurol Neurosurg Psychiatry.

[21] Hannon N, Sheehan O, Kelly L, Marnane M, Merwick A, Moore A (2009). Stroke associated with atrial fibrillation - Incidence and early outcomes in the North Dublin population stroke study. Cerebrovasc Dis.

[22] Hardie K, Jamrozik K, Hankey GJ, Broadhurst RJ, Anderson C (2005). Trends in five-year survival and risk of recurrent stroke after first-ever stroke in the Perth community stroke study. Cerebrovasc Dis.

[23] Mohan KM, Wolfe CDA, Rudd AG, Heuschmann PU, Kolominsky-Rabas PL, Grieve AP (2011). Risk and cumulative risk of stroke recurrence: a systematic review and meta-analysis. Stroke.

[24] Lovett JK, Coull AJ, Rothwell PM (2004). Early risk of recurrence by subtype of ischemic stroke in population-based incidence studies. Neurology.

[25] Mohan KM, Crichton SL, Grieve AP, Rudd AG, Wolfe CDA, Heuschmann PU (2009). Frequency and predictors for the risk of stroke recurrence up to 10 years after stroke: the South London stroke register. J Neurol Neurosurg Psychiatry.

[26] Moro CHC, Gonçalves ARR, Longo AL, Fonseca PG, Harger R, Gomes DB (2013). Trends of the incidence of ischemic stroke thrombolysis over seven years and one-year outcome: a population-based study in Joinville, Brazil. Cerebrovasc Dis.

[27] Martins SCO, Pontes-Neto OM, Alves CV (2013). Past, present, and future of stroke in middle-income countries: the Brazilian experience. Int J Stroke.

[28] Hata J, Tanizaki Y, Kiyohara Y, Kato I, Kubo M, Tanaka K (2005). Ten year recurrence after first ever stroke in a Japanese community: the Hisayama study. J Neurol Neurosurg Psych.

[29] Azarpazhooh MR, Nicol MB, Donnan GA, Dewey HM, Sturm JW, Macdonell RA (2008). Patterns of stroke recurrence according to subtype of first stroke event: the North East Melbourne Stroke Incidence Study (NEMESIS). Int J Stroke.

